# Determination of Methoxyfenozide Residues in Water and Soil by Liquid Chromatography: Evaluation of its Environmental Fate Under Laboratory Conditions

**DOI:** 10.5487/TR.2008.24.3.207

**Published:** 2008-09-01

**Authors:** Jeong-Heui Choi, M. I. R. Mamun, Eun-Ho Shin, Hee Kwon Kim, A. M. Abd El-Aty, Jae-Han Shim

**Affiliations:** 18 Natural Product Chemistry Laboratory, Division of Applied Bioscience and Biotechnology, College of Agriculture and Life Science, Chonnam National University, 300 Yongbong-dong, Buk-gu, Gwangju, 500-757 Korea; 28 Department of Chemistry, University of Dhaka, Dhaka, 1000 Bangladesh; 38 Vegetables Crops Experiment Station, Jeonnam Agricultural Research & Extention Service, Jeollanam-do, 542-821 Korea; 48 Department of Veterinary Pharmacology and Toxicology, College of Veterinary Medicine, Konkuk University, 1 Hwayang-dong, Kwangjin-gu, Seoul, 143-701 Korea; 58 Department of Pharmacology, Faculty of Veterinary Medicine, Cairo University, 12211-Giza Korea

**Keywords:** Insecticide, Fate, Method validation, Residue analysis, Experimental conditions

## Abstract

Pesticide residues play several key roles as environmental and food pollutants and it is crucial to develop a method for the rapid determination of pesticide residues in environments. In this study, a simple, effective, and sensitive method has been developed for the quantitative analysis of methoxyfenozide in water and soil when kept under laboratory conditions. The content of methoxyfenozide in water and soil was analyzed by first purifying the compound through liquid-liquid extraction and partitioning followed by florisil gel filtration. Upon the completion of the purification step the residual levels were monitored through high performance liquid chromatography (HPLC) using a UV absorbance detector. The average recoveries of methoxyfenozide from three replicates spiked at two different concentrations and were ranged from 83.5% to 110.3% and from 98.1% to 102.8% in water and soil, respectively. The limits of detection (LODs) and limits of quantitation (LOQs) were 0.004 vs. 0.012 ppm and 0.008 vs. 0.024 ppm, respectively. The method was successfully applied to evaluate the behavioral fate of a 21% wettable powder (WP) methoxyfenozide throughout the course of 14 days. A first-order model was found to accurately fit the dissipation of methoxyfenozide in water with and a DT_50_ value of 3.03 days was calculated from the fit. This result indicates that methoxyfenozide dissipates rapidly and does not accumulate in water.

## INTRODUCTION

Methoxyfenozide [3-methoxy-2-methylbenzoic acid 2-(3,5-dimethylbenzoyl)-2-(1,1-dimethylethyl) hydrazide], a diacylhydrazine insecticide, was originally discovered and characterized by Rohm and Hass Company, Italy (Wing, 1988; Wing *et al.*, 1988). It was first announced as the most efficacious member of the diacylhydrazine class by Le *et al.* in 1996. Its efficacy against beet armyworm, European corn borer, and codling moth is particularly noteworthy (Carlson *et al.*, 2001). In addition, methoxyfenozide has been shown to act against a wide range of lepidopteron pests of cotton, corn, and other major agronomic crops (Oberlander *et al.*, 1998; Olsza and Pluciennik, 1998; Smagghe *et al.*, 1998, 1999, 2000, 2001; Adamczyk *et al.*, 1999; Adel and Sehnal, 2000; Trisyono *et al.*, 2000). As a consequence of its ubiquitous use, the analyte is frequently

†The two authors contributed equally to this paper

**Table 1.** Physico-chemical properties of the soil used for experiments

| Sample | pH (1 : 50) | Organic matters (g/kg) | Cation exchange capacity (cmol+/kg) | Distribution of particle size (%) | | | Soil texture |
|---|---|---|---|---|---|---|---|
| | | | | Clay | Slit | Sand | |
| Soil | 5.3 | 18.3 | 12.9 | 36.0 | 50.9 | 12.1 | SiCl |

found in soil and other environmental matrices, constituting an animal and human health hazard. Moreover, there have been increasing concerns about the negative impacts of pesticides on the ecological communities, antibacterial resistance, and insecticidal resistance at low-levels of exposure (Halling-Sørensen *et al.*, 1998; Daughton and Ternes, 1999; Relyea and Hoverman, 2006). Therefore, studies on the environmental fate of methoxyfenozide can provide first-hand data that will be highly relevant in evaluating the potential risks of this pesticide. Furthermore, the results of the environmental fate of this compound will provide a guide for the field application and use of methoxyfenozide as a pesticide. Despite the importance of understanding and quantifying these factors, no studies have yet been conducted for this purpose. Therefore, the aim of the current investigation is to regularly monitor the residues through analytical method that combine short analysis time, sufficient selectivity, and sensitivity in order to better understand the environmental fate of methoxyfenozide in water and soil when kept under laboratory conditions.

## MATERIALS AND METHODS

***Solvents and reagents.*** Analytical standard of methoxyfenozide (100% purity) and the formulated product of 21% WP were kindly supplied by Dow AgroSciences (Seoul, Republic of Korea). Acetonitrile, dichloromethane, *n*-hexane, acetone, and anhydrous sodium sulfate used in the experiments were of pesticide residue (PR) analytical grade and were purchased from Merck KGaA (Darmstadt, Germany). Florisil gel (60~100 mesh, PR grade) was provided by Sigma (Missouri, USA) and Celite 545 (used for filtration) was obtained from Yakuei Pure Chemical Co. Ltd. (Kyoto, Japan).

***Treatment of water and soil with methoxyfenozide.*** The trials were carried out with air-dried soil in the shade which was friendly supplied from the Jeollanam-do Agricultural Research & Extension Services, located at Naju, Republic of Korea. This soil was put into experimental water baths, and the water was poured. Formulated product of methoxyfenozide diluted 5000 times was applied to 15 experimental water baths (0.04 $m^2$) of the same size at the same rate of a.i. 0.008 kg/10a. To avoid any variation in the concentration of applied pesticide (due to the evaporation of water), the experimental water bath was refilled with supplementary water every 3 days. The experimental baths treated with the pesticide were kept in the shade to protect decomposition of pesticide against solar light.

***Physico-chemical properties of the soil.*** The physical and chemical properties (Table 1) of the experimental soil were determined as follows: pH was determined with a Seven Easy model pH meter (Mettler Toledo, USA), organic matter content was determined by the Walkley and Black method as described by Allison (1965), and cation exchangeable capacity (CEC) was assayed by the 1 *N* $CH_3COONH_4$ method (RDA, 1988).

***Sampling.*** The water sample was collected using a beaker from three experimental baths at day 0 (2 h) after application of methoxyfenozide such that no soil residue came along from the bottom of the water bath. Later, the rest of the water was drained out, and the remaining soil was thoroughly mixed in a plastic bottle and collected. Similarly, samples were collected at 1, 3, 7, and 14 days from the other twelve experimental baths after pesticide application. For each time point 3 experimental baths were used.

**Sample extraction procedure and purification.**

***Water sample***: Water samples (100 ml) were mixed with acetone (100 ml) and distilled water (50 ml) and then filtered through Celite under pressure using a Buckner funnel. The filtrate was transferred to a separatory funnel (500 ml) and 50 ml of a saturated NaCl solution was added. The mixture was partitioned twice with dichloromethane (100 ml × 2). The combined dichloromethane layers were completely evaporated and reconstituted with 5 ml of *n*-hexane. The extract was purified by a glass column (11 mm × 400 mm) filled with florisil gel pre-activated at 110°C for 15 h. The column was washed with 50 ml of dichloromethane/*n*-hexane/acetonitrile (50/48.5/1.5, v/v/v) and eluted with another 50 ml of the same solvent mixture. The effluent was evaporated under pressure and re-dissolved in 2 ml of acetonitrile and analyzed using HPLC-UVD.

***Soil sample***: Soil samples were taken from the bottom of the experimental water bath after removing the water. Approximately 50 g of the soil sample was placed into a conical flask and 100 ml of acetone and 50 ml of distilled water were added. The mixture was shaken by a mechanical shaker (Eyela multishaker MMS, Japan) for 30 min followed by pressurized filtration. The filtrate was then placed into a separatory funnel (500 ml), partitioned, purified, and analyzed by a similar method as described above for the water samples.

***HPLC equipment and chromatographic conditions.*** A High performance liquid chromatograph (Kontron Instruments 322 System) connected to a UV-VIS detector was used to identify methoxyfenozide. Identification of the target analyte was carried out on a Novapak $C_{18}$ column (3.9 × 300 × 150 mm, Waters). A ternary mixture of water, acetonitrile, and methanol (6 : 3 : 1, v/v/v) was used as the mobile phase with a flow rate of 0.5 ml/min. Under these conditions the retention time for methoxyfenozide was 18.5 min.

***Method performance***: The recovery experiments were carried out by spiking blank water or soil samples at two different concentrations. The control water sample was spiked at 0.04 and 0.1 ppm levels and the soil samples were spiked at 0.08 and 0.2 ppm levels. The resulting samples were mixed and then allowed to stand for 15 min prior to extraction. The samples were then submitted to extraction and analysis using the same technique described previously. The relative recovery (extraction efficiency) of the analyte from water and soil was determined by comparing the areas of extracted analyte to that of the non-extracted pure standard (prepared in acetonitrile), which represents 100% recovery.

The precision of this method was expressed as the repeatability (RSD%) of the recovery determinations at the two different spiking levels.

The limit of detection (LOD) was defined as the analyte concentration that generated a response three times greater than the noise level of the detection system. The limit of quantification (LOQ) was defined as the minimum analyte concentration that was equivalent to ten times the noise, including the instrument noise and the background signal contributed by the matrix blank.

***Preparation of stock solution and standard curve***: A stock solution of methoxyfenozide of 100 ppm was prepared by dissolving 10 mg of the standard reference sample into 100 ml of acetonitrile. The stock solution was serially diluted by acetonitrile to obtain concentrations of 0.2, 0.5, 1.0, 2.0, and 5.0 ppm. Ten μl of each concentration was injected into the HPLC column and the calibration curves were prepared based on the peak height of each chromatogram.

***Data analysis***: The dissipation half-lives ($DT_{50}$) were calculated using a first-order dissipation model (Hu and Coats, 2007). Equation (1) describes the dissipation kinetics, and equation (2) was used to calculate dissipation half-live:

$$C_t = C_0 \text{ X } e^{(-kt)} \quad (1)$$

$$DT_{50} = 0.693/k \quad (2)$$

Where $C_0$ and $C_t$ are the concentration of the analyte at time 0 and time t (days), and k is a first-order rate constant determined from the slope of the test substance dissipation curves.

## RESULTS AND DISCUSSION

***Calibration and linearity.*** The standard calibration curves were determined by injecting the standard solutions of methoxyfenozide into the HPLC-UV system at five different concentration levels (ranging from 0.2~5.0 ppm). From these injections the calibration curves were found to be linear with a correlation coefficient ($R^2$) > 0.998 for both matrices.

***Method performance.*** The average percent recoveries from three replicate experiments of methoxyfenozide at the two different concentration levels examined were found to be between 83.5 and 110.3% in water and ranged from 98.1 to 102.8% in soil. These recovery rates were satisfactorily high and highly reproducible, confirming the applicability of the method. Precision was calculated in terms of intra-day repeatability. The intra-day repeatability was examined in terms of the percentage relative standard deviation (RSD%) ($n$ = 3) at two concentration levels of the analyte in both water and soil. The overall intra-day variations (RSD) ranged from 2.5~12% in water and 1.6~8.3% in soil (Table 2). The current findings are consistent with the requirements of European Union Guidelines (Document SANCO/10476/2003, 2004).

**Table 2.** Spiking level (ppm), recovery (%), relative standard deviations, RSD (%), limits of detection (LOD, ppm), limits of quantification (LOQ, ppm), and instrumental detection limit (ng) obtained by HPLC analysis of methoxyfenozide in water and soil at two spiking levels (n = 3)

| Sample | Spiked conc. | Recovery | RSD | LOD | LOQ | IDL |
|---|---|---|---|---|---|---|
| Water | 0.04 | 110.3 | 2.5 | 0.004 | 0.012 | 2 |
| | 0.1 | 83.5 | 12 | | | |
| Soil | 0.08 | 98.1 | 1.6 | 0.008 | 0.024 | |
| | 0.2 | 102.8 | 8.3 | | | |

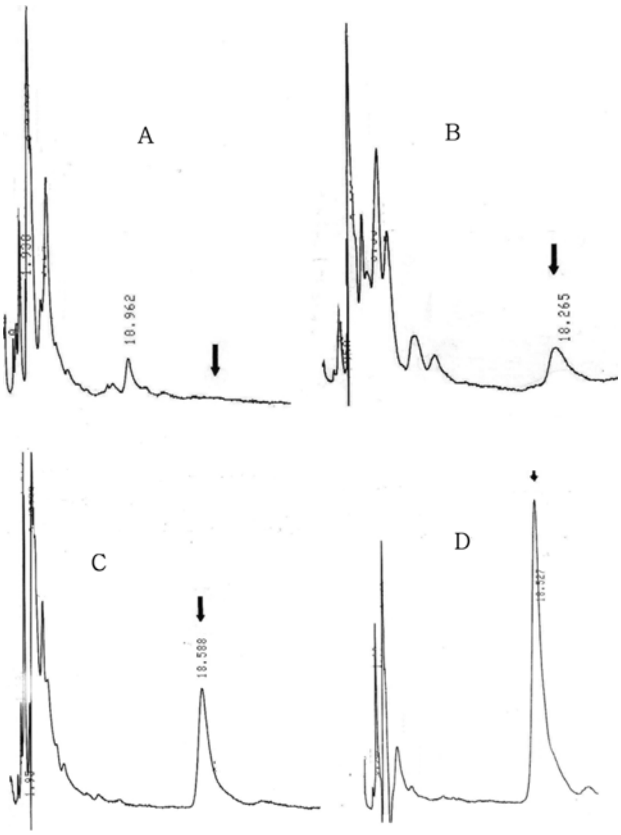

**Fig. 1.** Representative chromatograms of (A) blank, (B) methoxyfenozide standard, (C) fortified water sample, and (D) residual methoxyfenozide in the water sample at day 0.

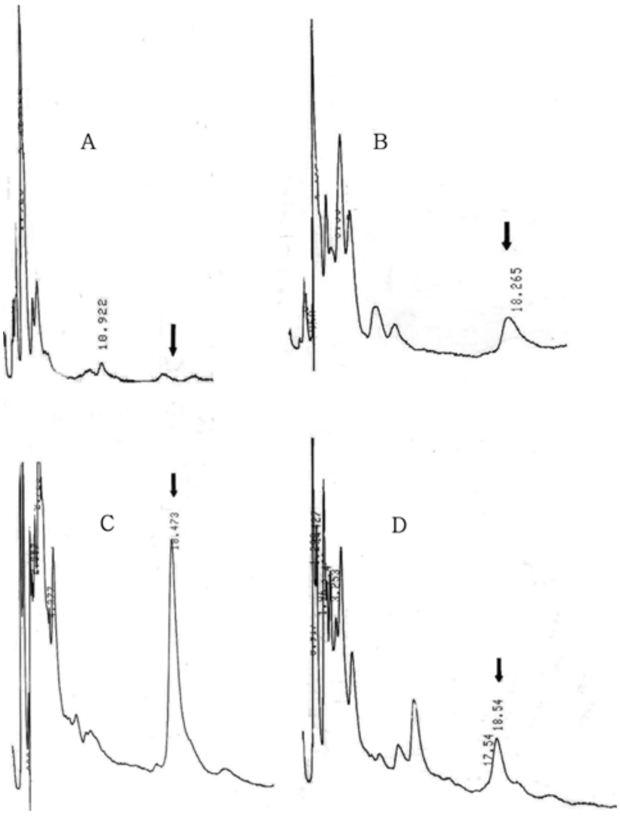

**Fig. 2.** Representative chromatograms of (A) blank, (B) methoxyfenozide standard, (C) fortified soil sample, and (D) residual methoxyfenozide in the soil sample at day 1.

The limits of detection (LOD) and limit of quantification (LOQ) were calculated as three and ten times the signal-to-noise ratio, respectively. The LOD and LOQ were found to be 0.004 and 0.012 ppm for water, and 0.008 and 0.024 ppm for soil, respectively (Table 2). These results clearly indicate that this method is sufficiently sensitive to allow the measurement of the analyte in both water and soil. It is worth noting that the MRL of this analyte has not yet been established by the Korea Food and Drug Administration (KFDA, 2005).

Selectivity was assessed by comparing the chromatograms of blank water or soil with those from the spiked samples. Endogenous peaks at the retention time of the analyte were not observed in any of the evaluated samples, indicating that there was no obvious direct interference at the expected retention time. Representative chromatograms of blank and spiked samples are shown in Figs. 1 and 2.

***Dissipation pattern of methoxyfenozide in water and soil.*** The dissipation pattern of a commercial product of methoxyfenozide (21% WP) was evaluated following its application at a rate of a.i. 0.0084 kg/10a to water bath containing soil at the bottom. The residual amounts in the water and soil were measured at 0, 1, 3, 7, and 14 days after introduction of the commercial product. It is worth noting that no quantifiable residues were observed in the control samples. The result from these experiments showed that the amounts of methoxyfenozide gradually decreased from the water with a half-life of 3.03 days (Table 3). Since fluorescent light (rather than solar light) was used as the irradiation source in this study, photodegradation might have had an effect on the rate of chemical degradation in this environment (Hu and Coats, 2007). In the soil samples, an increase in the residual levels was observed until day 7 (Table 3). It is plausible that this occurred because of absorption of the insecticide at the surface of the soil lying at the bottom of the water bath. This explanation is supported by the fact there was a sharp decrease in the amount of methoxyfenozide at day 7 relative to previous days in the water samples. However, the dissipation in water was quite acceptable and seemed to follow first order kinetics (Fig. 3).

**Table 3.** Amount of methoxyfenozide residues in experimental water and soil

| Sample | Days after application | Residues (ppm) | | | | Remark |
|---|---|---|---|---|---|---|
| | | 1R | 2R | 3R | Average | |
| Water | blank | <0.004 | <0.004 | <0.004 | <0.004 | 3.03 days ($R^2$ = 0.9848) |
| | 0 | 0.257 | 0.243 | 0.240 | 0.247 | |
| | 1 | 0.216 | 0.228 | 0.216 | 0.220 | |
| | 3 | 0.157 | 0.143 | 0.140 | 0.147 | |
| | 7 | 0.075 | 0.063 | 0.083 | 0.047 | |
| | 14 | 0.006 | 0.008 | 0.016 | 0.010 | |
| Soil | blank | <0.008 | <0.008 | <0.008 | <0.008 | ND |
| | 0 | 0.012 | 0.009 | 0.014 | 0.012 | |
| | 1 | 0.039 | 0.017 | 0.012 | 0.023 | |
| | 3 | 0.066 | 0.061 | 0.046 | 0.058 | |
| | 7 | 0.093 | 0.090 | 0.090 | 0.091 | |
| | 14 | 0.012 | 0.009 | 0.009 | 0.010 | |

1R, 2R, 3R denote the number of replicates (n = 3).
ND: not determined.

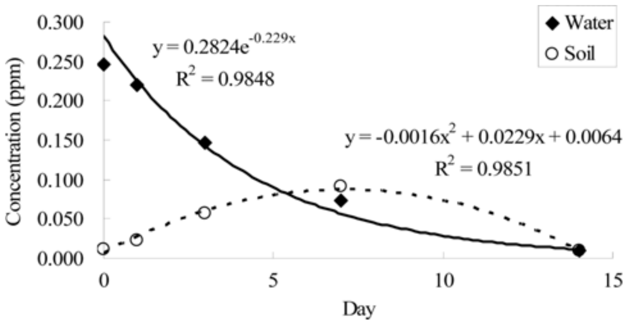

**Fig. 3.** Behavioral fate of methoxyfenozide in water and soil samples.

In the current investigation, the half-life of methoxyfenozide in water was found to be 3.03 days, which is far below the value reported in the WHO Report 2003 (2004). In this report the hydrolytic stability of methoxyfenozide in sterile buffer solutions at different pH was evaluated. From this work, methoxyfenozide was found to be stable for a 30-day test period and had a calculated half-life of 600 days at pH 5, 1600 days at pH 7 and 700 days at pH 9.

In the WHO report, the persistency of methoxyfenozide in soil was also determined from the aerobic degradation of radio-labeled methoxyfenozide in four different soil samples at a concentration of a.i. 0.075 kg/10a over one-year period. These results demonstrated that methoxyfenozide is very persistent in soil, where 59~75% of the applied dose remained after one year. Calculated first-order half-lives in this experiment ranged from 340 to 1100 days, depending on the soil. This work supports our findings that residual amounts of methoxyfenozide remain in the soil but in this experiment it was not possible to calculate the half-life because of the short experimental time periods (Fig. 3).

## CONCLUSION

A sensitive method based on LLE followed by SPE cleanup and final analyte determination by HPLC-UVD has been developed for the analysis of methoxyfenozide in water and soil. Methoxyfenozide has been efficiently determined with or without SPE cleanup using LC-MS/MS by other scientists (Choi *et al.*, 2001; Hall *et al.*, 2004; Hiemstra and Kok, 2007; Wang and Wotherspoon, 2007). However, LC-MS/MS is not familiar to every laboratory and needs professional knowledge and experiences to operate it. The developed method could achieve good extraction efficiencies such as percent recoveries and RSD using general HPLC-UVD and cleanup procedure, preparative glass column chromatographic separation, without using commercial SPE cartridges. The recoveries achieved in both matrices were within an acceptable range with RSDs of ≤ 12%. The analyte was found to dissipate rapidly and did not accumulate in water. Because of the short time period of sampling conducted in this experiment (14 days after application), we were unable to determine the dissipation half-life in soil. This means that the analyte might persist in the soil for a longer duration, a hypothesis that will require further investigation in addition to the environmental fate of the analyte under field conditions. Based upon the WHO report and the results presented here it appears that the analyte may be adversely affecting the soil fauna and therefore its use as a pesticide in vegetable crops should be avoided.

## ACKNOWLEDGEMENTS

This study was mainly supported by Dow Agro Science, Republic of Korea. We are also thankful to Institute of Agricultural Science and Technology, Chonnam National University for partially support to the study.
